# Evaluation of genomic selection to improve survival of eastern oysters infected with *Perkinsus marinus*

**DOI:** 10.3389/fgene.2026.1821653

**Published:** 2026-06-19

**Authors:** Thomas A. Delomas, Dina A. Proestou, Jessica M. Small

**Affiliations:** 1 United States Department of Agriculture, Agricultural Research Service, National Cold Water Marine Aquaculture Center, Kingston, RI, United States; 2 Aquaculture Genetics and Breeding Technology Center, Virginia Institute of Marine Science, William & Mary, Gloucester Point, VA, United States

**Keywords:** *Crassostrea*, dermo, disease challenge, genetic improvement, GWAS, marker assisted selection, selective breeding

## Abstract

Dermo disease is a chronic disease of eastern oysters infected by the protozoan parasite *Perkinsus marinus*. This disease often causes mortality on farms at the end of the grow-out cycle and has been identified by producers as a top concern. We here assessed the potential for marker-assisted selection and genomic selection to improve survival to *P. marinus* infection in a laboratory disease challenge. Three year classes of oysters from a family-based breeding program were challenged and genotyped with a 66k single nucleotide polymorphism (SNP) array. Six SNPs were identified through a genome-wide association study as significantly influencing survival, but none explained more than 4% of the total genetic variance for survival. This suggests that marker-assisted selection is not a viable option. Genomic selection was estimated to yield a 9% relative increase in accuracy compared to pedigree-based selection, and using a subset of 3,500 SNPs was found to give comparable accuracy to that with the full panel.

## 1 Introduction

Dermo disease is a chronic disease caused by the protozoan parasite *Perkinsus marinus* in eastern oysters *Crassostrea virginica*. *P. marinus* is widespread in eastern oyster growing regions (US Atlantic Coast and Gulf of Mexico) and often causes mortality toward the end of the grow-out phase (Craig et al., 1989; Ford and Smolowitz, 2007). Reflective of the disease’s impact, producers consistently rank improving resistance to dermo as a top priority when asked about traits to include in a selective breeding program (Rheault, 2018).

There are few genetically improved strains of eastern oysters available to producers, and none have been selected specifically for resistance to dermo disease. A small number of strains have been selected for growth and field survival and others have been opportunistically selected for survival to disease outbreaks (Allen Jr et al., 2021; Guo et al., 2008; Proestou et al., 2016; Rawson et al., 2010). While selecting for field survival or survival during an outbreak can be effective, it is unpredictable and the target phenotype (survival to the disease of interest) is confounded by other stressors.

To address the issues inherent in relying on natural outbreaks, oysters from a family-based selective breeding program were subject to laboratory disease challenges with *P. marinus.* It was shown that differences in survival rates exist and gene expression patterns vary between families with high and low survival in response to the parasite (Proestou et al., 2019; Proestou and Sullivan, 2020), implying the existence of genetic variance for survival to *P. marinus* infection. Further application of this challenge methodology to 109 full-sibling families from the same breeding program demonstrated that survival is moderately heritable with a narrow-sense heritability of 0.14 ± 0.03 on the observed scale (0.24 ± 0.05 underlying liability, which assumes an underlying continuous trait) (Proestou et al., 2025). This implies that selection can improve the rate of survival.

The efficacy of selection for a disease challenge phenotype is limited when breeding values are estimated based on pedigree relationships (Houston et al., 2020; Saura et al., 2019). This is primarily because the selection candidates are typically not challenged with the disease (doing so would be a biosecurity risk) and are therefore not phenotyped. This prevents pedigree-based methods from exploiting within-family genetic variation.

This drawback can be addressed by using molecular markers to inform the calculation of breeding values. Marker-assisted selection utilizes within-family genetic variance and is highly cost-effective as it requires genotyping one or a few loci (Meuwissen et al., 2013). However, this strategy is only applicable when variation in the trait of interest is controlled by one or a small number of loci with large effect sizes and known markers are in strong linkage disequilibrium with the causal allele(s).

Genomic selection requires genotyping a larger number of loci than marker-assisted selection, but it is applicable to traits with oligogenic or polygenic architectures (Habier et al., 2011; Meuwissen et al., 2013). Over the past few decades, genomic selection has proven to be a robust method for estimating breeding values for a variety of traits in oysters and other species. For example, the accuracies of breeding values for growth traits and survival to a viral disease challenge in Pacific oysters *Crassostrea gigas* were improved by the use of genomic selection compared to pedigree-based methods (Gutierrez et al., 2020; Gutierrez et al., 2018; Jourdan et al., 2023). Genomic selection increased accuracy in selecting eastern oysters for growth traits (Marín-Nahuelpi et al., 2025), and it has been evaluated for increasing low-salinity tolerance (McCarty et al., 2022; Schwartz et al., 2026).

In order to inform eastern oyster selective breeding programs, we here assessed the applicability of marker-assisted and genomic selection for improving survival to a disease challenge with *P. marinus*. We challenged eastern oysters from a family-based breeding program and first performed a genome-wide association study (GWAS) to identify candidate loci for marker-assisted selection. We then compared the accuracy of genomic and pedigree-based estimated breeding values to assess the potential benefit from utilizing genomic selection.

## 2 Materials and methods

### 2.1 Oyster source and disease challenge

This report is a follow-up study to the work presented by Proestou et al. (2025) and analyzes samples from the same disease challenge. Juvenile oysters (1 year old) were obtained from the Aquaculture Genetics and Breeding Technology Center family-based oyster breeding program at Virginia Institute of Marine Science (Allen Jr et al., 2021) in 2020, 2021, and 2022. A total of 5,805 oysters derived from 109 full-sibling families (approx. 40–60 individuals per family) were challenged with an injection of *P. marinus* standardized by estimated meat weight as described by Proestou et al. (2019) and maintained in flow-through raceways at a temperature and salinity of 21 °C–24 °C and 30–35 ppt, respectively. Each family was represented by two or three replicates of 20 oysters kept in baskets and replicates were distributed between six tanks (multiple families per tank with no more than one replicate of a given family per tank). Oysters were maintained for 45 days, and survival was recorded daily. Additional details of the disease challenge can be found in Proestou et al. (2025).

### 2.2 Genotyping and filtering

Mantle tissue samples were collected from challenged oysters either on the date of death or at the end of the challenge and stored in 95% ethanol. Mantle tissue samples from the parents of the challenged individuals were collected at the time of spawning and stored in 95% ethanol. DNA was extracted from approximately 5 mg of preserved tissue using QuickGene DNA tissue kits (Pereira et al., 2011) and then genotyped on a 66k SNP array (Guo et al., 2023) by the Center for Aquaculture Technologies.

The genotypes were first filtered to remove loci with a high rate of Mendelian incompatibilities (MIs). A subset (n = 257) of the potential parents of the challenged oysters were genotyped on either the 66k array or a larger array that contained all loci on the 66k array (Guo et al., 2023) as part of a separate experiment. In order to reduce the potential for pedigree recording or laboratory errors to inflate the number of MIs, we did not consider the recorded parentage. Instead, we inferred parentage using the genotypes.

Inferring parentage in bivalves is sometimes reported to be challenging when not using loci specifically selected for parentage inference in the target population (Massault et al., 2021; Wan et al., 2017). This is presumably due to null alleles caused by high rates of polymorphism and presence-absence variation, which have been found to be prevalent in bivalves (Guo et al., 2023; Hedgecock et al., 2004; Li et al., 2003; Reece et al., 2004; Sauvage et al., 2007; Zhang and Guo, 2010). Parentage inference in the current study is additionally complicated by the interrelated nature of the population (Anderson and Garza, 2006; Olsen et al., 2001). We therefore used a multi-step Mendelian exclusion process that 1) identified preliminary parent-offspring trios, 2) used these trios to identify high-confidence loci, and 3) used these loci to then infer trios. We started by selecting only loci with minor allele frequency (MAF) in the range of 0.45–0.50 in the parents and identified potential parent-offspring pairs by counting pairwise MIs. Only highly polymorphic loci were selected in order to maximize informativeness while increasing the efficiency of computation. Based on visual observation of the distribution of MIs, pairs with fewer than 7.5% of MIs were considered for trio assignment as this was partially into the second peak of the bimodal MI distribution (Supplementary Material Figure S1). This threshold was set purposefully high (overlapping the second peak) in order to minimize the false negative rate at this step. Preliminary parent-offspring trios were then inferred by accepting, for each offspring, the trio with fewest MIs among those with a maximum of 9% MIs. This threshold was chosen based on visual examination of the MI distribution and separated the first and second peak of the bimodal distribution (Supplementary Material Figure S2). The preliminary trios were used to count the number of MIs for each locus and only those loci with MIs in less than 2% of trios were used in the following steps. The parent inference steps were repeated, but with thresholds for parent-offspring pairs and trios of 5% and 2%, respectively (Supplementary Material Figures S3,S4). This process yielded no offspring with assignments to more than one trio, which indicates a low rate of incorrect assignment. The parentage inference process was performed using custom C++ functions and R scripts linked through the Rcpp package (Eddelbuettel and Francois, 2011).

The inferred trios were then used to count the number of MIs for all loci, and loci displaying more than 2% MIs were removed. For a locus with minor allele frequency of 0.21 (the mean in this study, Section 3) this corresponds to an equivalent genotyping error rate of approximately 2.5% (Saunders et al., 2007). Using genotypes of only the challenged individuals, loci and individuals with completeness rates lower than 90% as well as loci with MAF less than 1% were removed. Samples with duplicate genotypes were identified by calculating the percentage of genotypes identical by state using PLINK (Chang et al., 2015). In duplicate pairs with greater than 95% identity, the sample with lower genotyping completeness was removed. Missing genotypes were imputed using AlphaImpute2 (Whalen and Hickey, 2020) and were informed by the genotypes of inferred parents.

### 2.3 Repeatability evaluation

When filtering for MIs, we eliminated a large fraction of loci (see results). To verify that this was not a problem with panel repeatability, we genotyped a set of 34 samples twice (on two different chips) and compared genotypes between the replicates. Of these 34 samples, 12 were from oysters that were part of the challenge described here and the other 22 were from two natural-origin populations, one in Rhode Island, USA (n = 11 oysters) and one in Maine, USA (n = 11). Genotype concordance was calculated between replicates with missing genotypes being excluded.

### 2.4 GWAS

A GWAS was performed to assess the genetic architecture of survival in the disease challenge and determine the feasibility of marker-assisted selection. A linear mixed model fit by GEMMA software (Zhou and Stephens, 2012) was used to estimate each SNP effect on the response variable of survival at the end of the experiment (0 or 1). Other fixed effects were year class and tank, and the model included a random effect controlling for relatedness as estimated by genotypes. Each SNP was evaluated with a likelihood ratio test as implemented in GEMMA and statistical significance was defined at a Bonferroni corrected (for the total number of SNPs assessed, 33,148) **α** = 0.05 (p < 1.5e-6).

To estimate the amount of genetic variation explained by a SNP with a statistically significant effect, we included the genotype (coded as 0, 1, or two allele copies) as a covariate in the animal model specified by Proestou et al. (2025). This model also contained fixed effects of year class and tank (nested within year class) as well as a random animal effect (based on the numerator relationship matrix calculated from the recorded pedigree) and a random replicate effect. The proportional reduction in the estimated variance of the animal effect when the SNP was included as a covariate was interpreted as the amount of genetic variation explained by that SNP (Boddicker et al., 2012).

### 2.5 Identification of candidate genes

To identify genes close to SNPs that were significantly associated with survival, we first assessed the distance at which the mean linkage between variants decayed to the background level. Pairwise linkage (*r* ^2^) was calculated using PLINK 1.9 and plotted within 1 kbp windows. Visual examination of linkage decay indicated that linkage approached the background level at 10 kbp. EntrezIDs for genes within 10 kbp of SNPs associated with survival were identified in the annotation for previous RefSeq assembly GCF_002022765.2 and the recently released RefSeq assembly GCF_053477285.1. Descriptive gene names and GO terms were identified using the R packages clusterProfiler (Wu et al., 2021) and AnnotationHub (https://doi.org/10.18129/B9.bioc.AnnotationHub). Results for both assemblies are presented as many laboratories are currently using the previous RefSeq assembly due to the very recent release of GCF_053477285.1. The locations of significantly associated SNPs in GCF_002022765.2 were taken from the annotation of the genotyping array (Guo et al., 2023) and locations in GCF_053477285.1 were identified by mapping the flanking sequence with BLAST (Johnson et al., 2008).

### 2.6 Evaluation of genomic selection

The potential benefit of genomic selection compared to pedigree-based selection was evaluated through 10-fold cross-validation to estimate accuracy following the method described by Legarra et al. (2008). In each iteration, 10% of individuals from each family replicate were masked (evenly distributed between replicates), as this represents the typical practice of a family-based aquaculture breeding program where full-siblings of the selection candidates are phenotyped (Allen Jr et al., 2021; Delomas et al., 2024; Gjedrem, 2010; Houston et al., 2020; Sonesson and Meuwissen, 2009). This breeding program structure increases the accuracy of breeding value estimation, which has motivated adoption by the aquaculture industry. The study here is specifically applicable to family-based aquaculture breeding programs. Other program structures with lower relatedness between the phenotyped animals and selection candidates will likely result in lower accuracy of genomic selection.

Genomic breeding values were estimated by applying an animal model with a genomic relationship matrix (i.e., GBLUP). Survival was modeled with a linear link function, an overall mean, and the fixed effects were year class and tank nested within year class. The use of a linear link function for modeling survival has previously been shown to be robust and offer computational advantages compared to threshold models in animal breeding. Comparing linear and threshold models for binary traits has consistently demonstrated no meaningful difference between the two approaches for breeding value estimation (Carlén et al., 2006; Holtsmark et al., 2009; Leeds et al., 2010; Ødegård et al., 2006; Ødegård et al., 2007). The random effects were a common environment effect designating the basket within a tank the animal was kept in (Proestou et al., 2025) and the additive genetic effect of the individual. The genetic relatedness matrix was calculated according to the first method of VanRaden (2008) and blended with the numerator relationship matrix (based on recorded pedigree) for computational stability with weights of 0.95 and 0.05, respectively (Fragomeni et al., 2017). These weights were chosen because they resulted in computational stability. Pedigree-based breeding values were calculated with the same animal model, except the numerator relationship matrix (based on recorded pedigree) and genetic group effects representing distinct founding populations of the VIMS breeding program (Allen Jr et al., 2021; Proestou et al., 2025) were used instead of the genomic relationship matrix. All animal models (genomic and pedigree) were fit using ASREML v4 (Gilmour et al., 2021). Accuracy was calculated as described by Legarra et al. (2008), $\frac{r\left(y,\hat{y}\right)}{H\Omega }$ , where $r\left(y,\hat{y}\right)$ is the correlation between observed and predicted phenotypes (corrected for fixed effects), H^2^ is the heritability, and Ω^2^ is the ratio of the genetic variance to the sum of the genetic and common environment variance. Both H^2^ and Ω^2^ were estimated from the BLUP model with all the data (Legarra et al., 2008).

To compare how panel-size affected the accuracy of genomic selection, we used a strategy similar to the LR method (Legarra and Reverter, 2018) of evaluation. We again applied 10-fold cross-validation in the manner described above but calculated genomic estimated breeding values (GEBVs) with 100, 500, 1,000, 2,000, 3,500, 5,000, 7,500, 10,000, or 20,000 SNPs. These SNPs were chosen randomly (without replacement) in each iteration from SNPs with MAF >0.05. No other criteria (e.g., distribution throughout the genome) were used in randomly selecting SNPs. The correlation of GEBVs for the masked individuals with those calculated using all SNPs and phenotypes was interpreted as a measure of relative accuracy. The underlying logic of this interpretation is that the GEBVs calculated using all information (all loci and phenotypes) are the most accurate available, and so the more correlated the estimates with a reduced panel are to the most accurate estimates, the higher the accuracy with the reduced panel.

## 3 Results

Survival to the challenge was 44, 73, and 77% in 2020, 2021, and 2022, respectively with an overall survival of 69%. Of the 5,805 oysters challenged, 5,720 had sufficient tissue recovered for genotyping. Preliminary parentage inference was performed using 5,051 loci with MAF >0.45 in the potential parents, and this was reduced for assignment of trios to 2,299 loci displaying low rates of MIs. A total of 5,010 parent-offspring trios were identified and 30,846 loci were excluded from further analyses with the entire array for displaying MIs in a high proportion (>2%) of these trios. Fourteen samples and 56 loci were removed for having less than 90% genotype completeness. An additional 1,812 loci with MAF less than 1% were removed. Nine pairs of duplicate genotypes were identified and the sample with higher genotype completeness was retained. After filtering, 5,697 samples and 33,148 loci remained for analysis. The mean (SD) MAF of these SNPs was 0.21 ± 0.14 and mean (SD) genotyping completeness for loci and individuals was 99.4% ± 1.0% and 99.4% ± 0.7%, respectively.

The repeatedly genotyped samples all had acceptable genotyping completeness with mean (SD) of 98.3% ± 0.7% and a range of 95.5%–99.2%. Repeatability of the array genotypes was high with a mean (SD) of 99.2% ± 0.31% of genotypes being concordant and no apparent difference between the three tested populations (Table 1). Of the genotypes that were non-concordant, the vast majority (>99%) were heterozygous - homozygous differences (Table 1).

**TABLE 1:** Concordance between repeated genotyping of the same samples.

Population	Mean concordance (%)	Concordance range (%)	Mean heterozygous - homozygous discordance (%)
VIMS	98.90	98.12–99.16	99.77
Maine	99.48	99.31–99.60	99.75
Rhode Island	99.31	99.11–99.46	99.70

The mean heterozygous - homozygous discordance is the mean percentage of discordant genotypes that were heterozygous in one replicate and homozygous in the other. VIMS, Virginia Institute of Marine Science.

**FIGURE 1:**
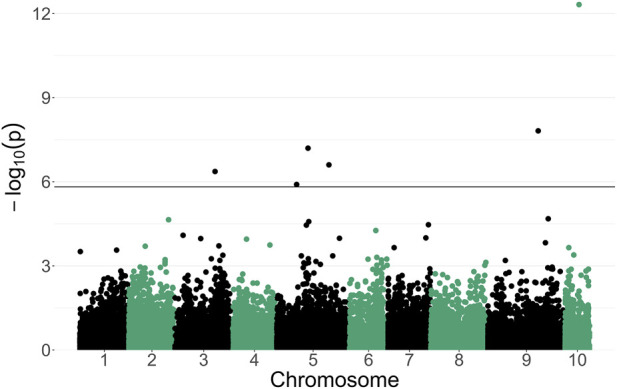
Manhattan plot of p-values for the association of SNPs with challenge survival with SNP locations from GCF_002022765.2. The horizontal line indicates the Bonferroni-corrected **α** = 0.05 (p < 1.5e-6).

A total of six SNPs with statistically significant effects on survival were identified (Figure 1). These SNPs were located on four different chromosomes in GCF_002022765.2 and four or five chromosomes in GCF_053477285.1 (one SNP mapped to two chromosomes, Table 2). Individually, each SNP explained between 0% and 4% of genetic variation for survival. There were 10 and 12 genes adjacent to these SNPs in GCF_002022765.2 and GCF_053477285.1, respectively, with nine genes in common between the two assemblies, one unique to GCF_002022765.2 and three unique to GCF_053477285.1. The GO terms associated with the genes were indicative of a broad range of cellular activities and did not obviously identify one specific pathway (Supplementary Material Tables S1, S2).

**TABLE 2:** SNPs with statistically significant effects on survival and EntrezIDs of adjacent genes (within 10 kbp) in two reference assemblies.

​	Genetic variance explained (%)	GCF_002022765.2		GCF_053477285.1	
SNP		Chromosome	Adjacent genes	Chromosome	Adjacent genes
AX-576895944	0	3, NC_035782.1	111125808, 111125809, 111125427	2, NC_136048.1	111125808, 111125809, 111125427
AX-575892659	2	5, NC_035784.1	111135424, 111133628, 111134691	5, NC_136051.1	111135424, 111133628, 111134691
AX-576898901	1	5, NC_035784.1	111132188, 111136759	5, NC_136051.1	111132188, 111136759
AX-576130134	2	5, NC_035784.1	111132202	7, NC_136053.1	111132202
AX-573987819	4	9, NC_035788.1	NA	2, NC_136048.1*	111127432, 111126930, 111126929
AX-574075882	3	10, NC_035789.1	111116417	8, NC_136054.1	NA

*The flanking sequence for this SNP also had a plausible alignment to Chromosome 1, NC_136047.1 in GCF_053477285.1

**FIGURE 2:**
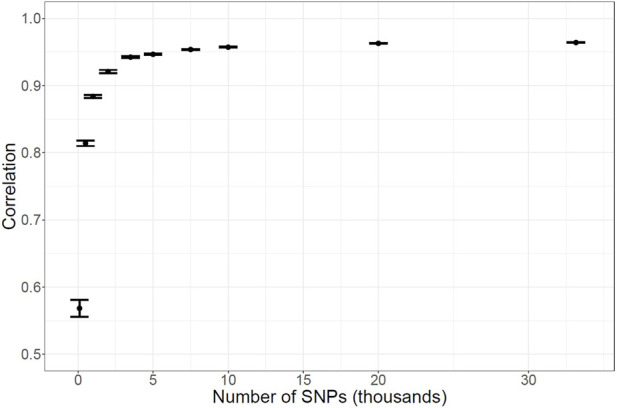
Mean correlation between GEBVs estimated for masked samples in the cross-validation routine with different numbers of SNPs and GEBVs estimated using all data (all loci and phenotypes for all individuals). Error bars represent standard error of the 10 cross validation iterations. For points with only one error bar visible, the standard error was miniscule and the 2 bars overlap at the scale of the figure.

Genomic selection using all 33,148 loci was estimated to be 9% more accurate than pedigree-based selection, with accuracies for PBLUP and GBLUP of 0.70 ± 0.04 and 0.76 ± 0.05, respectively. The correlation of GEBVs for masked individuals to those calculated with all information (all loci and phenotypes) was 0.96 when using all loci and only negligibly reduced by decreasing panel size to 3,500 loci (correlation of 0.94, Figure 2). Further decreases in panel size led to more substantial decreases in correlation of GEBVs. The estimated heritability of survival was slightly different from Proestou et al. (2025) at 0.15 ± 0.03 (compared to 0.14 ± 0.03) due to exclusion of samples that failed to genotype. Estimates of individual variance components are given in Supplementary Material Table S3.

## 4 Discussion

Survival to disease challenge with *P. marinus* has previously been found to have moderate heritability in two separate populations of eastern oysters (Proestou et al., 2025; Wang et al., 2025). The GWAS performed here only identified SNPs that explained a minor amount of the genetic variance and were not linked to genes with an obvious relation to a single pathogen-defense pathway. Similarly, a GWAS performed with a different population and *P. marinus* challenge methodology identified no statistically significant loci. These results demonstrate that survival to *P. marinus* challenge is a polygenic trait.

Marker-assisted selection is highly efficient when a single quantitative trait locus (QTL) is responsible for a majority of the genetic variance, but there are few commercially relevant traits where this is the case. Perhaps the best example in aquaculture, marker-assisted selection was used to breed resistance to infectious pancreatic necrosis virus in Atlantic salmon *Salmo salar* (Moen et al., 2015) after discovery of a QTL explaining 80%–100% of the genetic variance for this trait (Houston et al., 2008; Moen et al., 2009). The SNPs identified in the current study all explained 4% or less of the genetic variance, which is well below the range where marker-assisted selection is feasible. Most commercially relevant traits have polygenic (Yáñez et al., 2023) or oligogenic (Vallejo et al., 2017; Vallejo et al., 2010) architecture. While marker-assisted selection is not an option in these cases, genomic selection can be effective.

The current study indicates that genetic improvement for survival to *P. marinus* can be pursued through pedigree-based or genomic selection, but genomic selection is expected to be approximately 9% more accurate. A wide range has been reported for the improvement of accuracy when evaluating genomic selection for traits in other aquaculture breeding programs, and 9% is well within this range (Houston et al., 2020; Yáñez et al., 2023). While they did not evaluate pedigree-based selection, Wang et al. (2025) observed that genomically selected animals survived *P. marinus* challenge at a higher rate than phenotypically selected animals after one generation, illustrating the applicability of genomic selection for this trait.

Genomic selection is expected to increase accuracy, but it requires the expense of genotyping animals. The cost of genotyping is dependent on the number of loci targeted, and so we investigated whether smaller panels could perform as well as the SNP array used in the current study. A randomly selected set of 3,500 SNPs performed approximately equal to the full array (correlation of 0.94 vs. 0.96). This demonstrates that medium-density genotyping panels are sufficient for implementing genomic selection in family-based, eastern oyster breeding programs, similar to the conclusions from simulation studies (Delomas et al., 2023) and other aquaculture species (Kriaridou et al., 2020) including Pacific oysters *C. gigas* (Gutierrez et al., 2020). Further cost-savings could be achieved through an imputation strategy, with the high or medium-density panel being reserved for broodstock and a low-density panel being used on the phenotyped individuals and selection candidates (Delomas et al., 2023; Delomas et al., 2024).

Wang et al. (2025) also evaluated the effect of genotyping panel size on the accuracy of genomic selection for survival of *P. marinus* challenge. It was found that by ranking SNPs according to p-value from an association analysis (Luo et al., 2021; Zhao et al., 2021), approximately maximum accuracy could be achieved with a minimum of 10,000 top-ranked SNPs. The current study achieved approximately maximum accuracy with only 3,500 randomly selected SNPs. Comparison of these results is restricted by differences in the population and methodology used, but the sizeable discrepancy suggests that selecting SNPs randomly can out-perform ranking by p-value. This result can generally be expected, particularly when the GWAS used for ranking is not powerful enough to identify a sufficient number of QTLs to explain a large portion of the genetic variation. First, ranking based on p-value does not account for different magnitudes of the allele substitution effect for the identified QTLs. Second, if the GWAS used is not statistically powerful enough to identify most QTL, which will almost always be the case for polygenic traits, then many QTL will not be represented by SNPs with low p-values. Third, association analyses often identify multiple SNPs linked to the same QTL. By ranking SNPs according to p-value, the strongest QTL may be redundantly represented, leaving fewer SNPs to represent the rest of the genome. In contrast, selecting SNPs randomly, or weighted to be dispersed throughout the genome (Delomas et al., 2023), could more efficiently represent QTLs distributed across the genome. Reducing costs by using a medium-density panel may depend on choosing loci known to perform well in the target breeding program. We observed a large number of loci with a high (>2%) rate of MIs in the current study, but this was not due to repeatability issues with the array. Rather, it is likely due to population-specific issues with these loci, such as the presence of null alleles. Null alleles have been noted to cause non-Mendelian segregation in a large fraction of markers among both Pacific and eastern oysters (Guo et al., 2023; Hedgecock et al., 2004; Li et al., 2003; Reece et al., 2004). The use of a high-density array in the current study allowed these loci to be identified and removed while still ensuring that a sufficient number remained for analysis.

In this study, we compared marker panels of different sizes based on the correlation of GEBVs for masked individuals with those estimated from the full data (all phenotypes and markers). We chose this approach because it is grounded in logic, straightforward to compare different panels, and avoids some known sources of potential bias in the commonly used method to estimate accuracy through cross-validation (Legarra et al., 2008; Legarra and Reverter, 2017; Legarra and Reverter, 2018). We have included results for the more commonly used method (Legarra et al., 2008) in the Supplementary Material, and it supported an identical conclusion that 3,500 randomly chosen SNPs were sufficient for genomic selection in this case (Supplementary Methods and Supplementary Material Figure S5).

We show here that genomic selection can improve survival to *P. marinus* infection in eastern oysters. Survival is an informative phenotype, but it can be achieved through multiple underlying mechanisms, namely, resistance (reducing or eliminating pathogen load) and tolerance (lack of mortality at a given pathogen load). The implications of improving each are different, with resistance potentially causing herd immunity (Hulst et al., 2021) and tolerance potentially being more effective when a pathogen has high environmental prevalence (Bishop, 2012). Additionally, focusing on survival ignores the production potential of an animal after pathogen exposure. An animal’s potential may be affected by both the pathogen and the animal’s response. The trait examined in the current study, endpoint survival, does not allow us to differentiate between these potential mechanisms. Future examination of tolerance, resistance, and post-exposure production potential will allow refinement of genetic improvement strategies for reducing the impact of dermo disease.

## Data Availability

The datasets generated for this study can be found in the Ag Data Commons at https://doi.org/10.15482/USDA.ADC/32560086.v1.
