# Supplementary material for: Evaluation of genomic selection to improve survival of eastern oysters infected with Perkinsus marinus
Source: Front Genet. 2026 Jun 19;17:1821653. doi: 10.3389/fgene.2026.1821653 (PMC13327657; doi:10.3389/fgene.2026.1821653)
Supplement: Supplementary file 1 [file Supplementaryfile1.docx]

Supplementary Material

# Supplementary Methods

## Estimation of GBLUP accuracy with marker panels of reduced size

As described in the main text for comparing the accuracy of pedigree-based EBVs and GEBVs (see section 2.6), 10-fold cross validation was run and accuracy calculated following the method of Legarra et al. (2008). For each fold, accuracy was calculated using 100, 500, 1,000, 2,000, 3,500, 5,000, 7,500, 10,000, or 20,000 randomly chosen SNPs to inform estimation of GEBVs. For each iteration, the selected SNPs were the same ones used for the comparison of different sized marker panels described in the main text.

# Supplementary Figures and Tables

## Supplementary Figures

**Supplementary Figure 1.** Histogram of MIs in potential parent-offspring pairs with the initial set of 5,051 SNPs with MAF > 0.45. The vertical line represents 7.5% MIs, the threshold chosen for accepting a possible relationship in the first assessment of parent-offspring pairs.

**Supplementary Figure** **2.** Histogram of MIs in potential parent-offspring trios with the initial set of 5,051 SNPs with MAF > 0.45. The vertical line represents 9% MIs, the threshold chosen for accepting a possible relationship in the first assessment of parent-offspring trios.

**Supplementary Figure** **3.** Histogram of MIs in potential parent-offspring pairs with the reduced set of 2,299 SNPs used for parentage inference. The vertical line represents 5% MIs, the threshold chosen for accepting a possible relationship in the second assessment of parent-offspring pairs.

**Supplementary Figure 4.** Histogram of MIs in potential parent-offspring trios with the reduced set of 2,299 SNPs used for parentage inference. The vertical line represents 2% MIs, the threshold chosen for accepting a parent-offspring trio relationship.

**
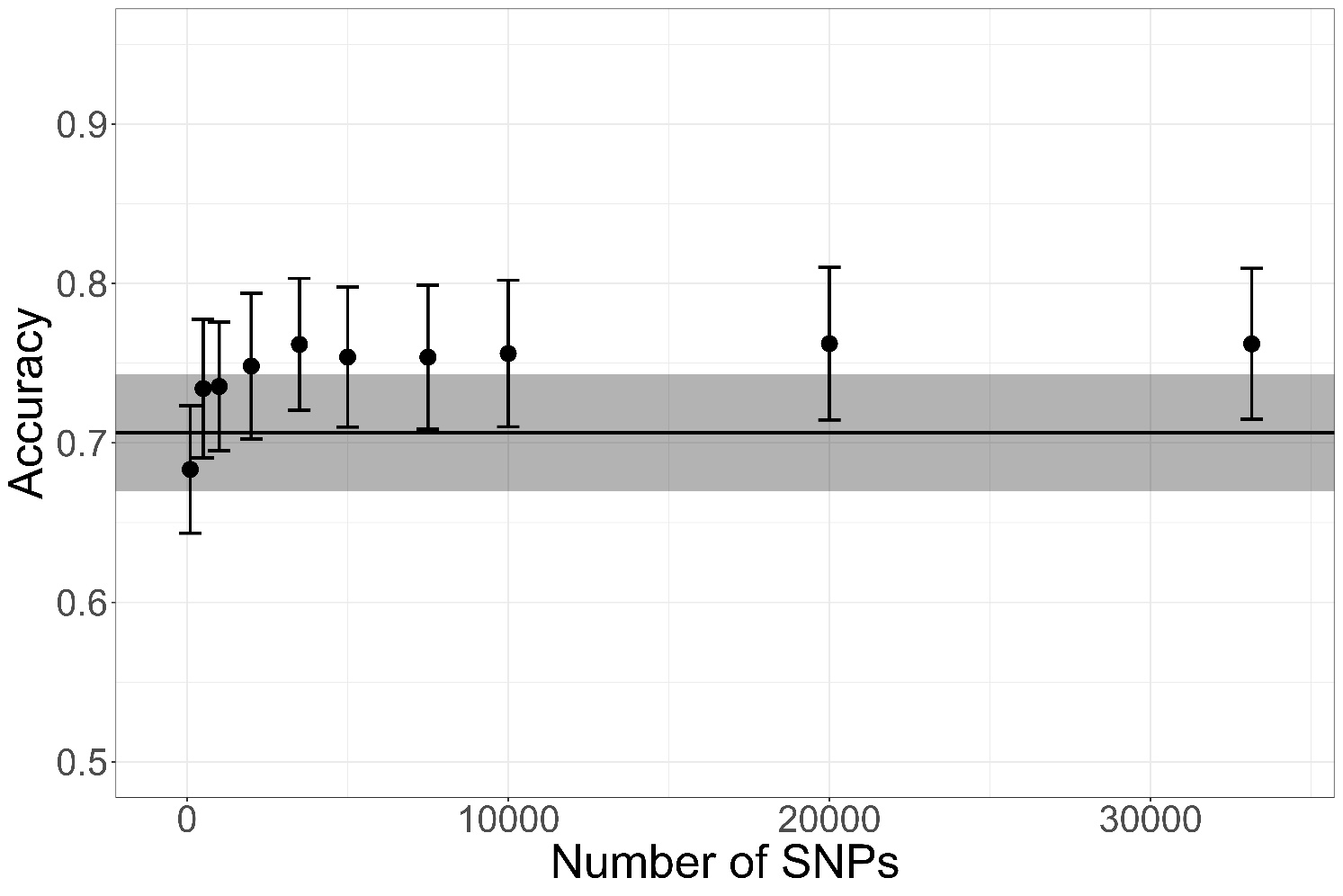
**

**Supplementary Figure 5.** Accuracy of GBLUP with marker panels of reduced size estimated by cross validation. The error bars represent SE. The horizontal line indicates the estimated accuracy of pedigree-based BLUP with the shaded area indicating the SE. Numbers of SNPs used were 100, 500, 1,000, 2,000, 3,500, 5,000, 7,500, 10,000, 20,000, or 33,148 (the full dataset after filtering).

## Supplementary Tables

| EntrezID | Gene name | Molecular function | Biological process | Cellular component |
| --- | --- | --- | --- | --- |
| 111125427 | ras-related protein Rab-14 | peptide receptor activity, GTPase activity, NAD+ nucleosidase activity, guanylate cyclase activity, protein kinase activity, protein binding, ATP binding, GTP binding | NA | extracellular space, Golgi apparatus |
| 111132188 | uncharacterized LOC111132188 | NA | endocytic recycling | NA |
| 111132202 | retinal guanylyl cyclase 2-like | NA | pore complex assembly, response to axon injury, cytolysis in another organism, phagosome maturation, proton transmembrane transport | phagocytic vesicle, pore complex, recycling endosome |
| 111133628 | uncharacterized LOC111133628 | NA | cGMP biosynthetic process, protein phosphorylation, monoatomic cation transport | NA |
| 111134691 | sterile alpha and TIR motif-containing protein 1-like | proton channel activity, channel activity | Golgi to endosome transport, signal transduction, receptor guanylyl cyclase signaling pathway | membrane, dendrite |
| 111135424 | type-1 angiotensin II receptor-associated protein-like | NA | NA | trans-Golgi network, plasma membrane |
| 111136759 | uncharacterized LOC111136759 | signaling adaptor activity | negative regulation of MyD88-independent toll-like receptor signaling pathway, intracellular signal transduction, angiotensin-activated signaling pathway, defense response to bacterium | NA |
| 111125808 | proteoglycan 4-like | NA | NA | NA |
| 111125809 | coiled-coil domain-containing protein 105-like | NA | NA | NA |
| 111116417 | uncharacterized LOC111116417 | NA | NA | NA |

**Supplementary Table 1.** Descriptive names and GO terms for genes adjacent to SNPs associated with survival in GCF_002022765.2.

| EntrezID | Gene name | Molecular function | Biological process | Cellular component |
| --- | --- | --- | --- | --- |
| 111125427 | ras-related protein Rab-14 | peptide receptor activity, GTPase activity, NAD+ nucleosidase activity, serine-type endopeptidase activity, guanylate cyclase activity, protein kinase activity, neuropeptide Y receptor activity, protein binding, ATP binding, GTP binding | NA | NA |
| 111126929 | allatostatin-A receptor-like | NA | cGMP biosynthetic process | trans-Golgi network, plasma membrane |
| 111126930 | fibrinolytic enzyme, isozyme C-like | NA | NA | extracellular space, Golgi apparatus |
| 111127432 | chymotrypsin-like serine proteinase | NA | NA | extracellular space, Golgi apparatus |
| 111132188 | uncharacterized LOC111132188 | NA | endocytic recycling | NA |
| 111132202 | retinal guanylyl cyclase 2-like | NA | pore complex assembly, response to axon injury, cytolysis in another organism, phagosome maturation, proton transmembrane transport | phagocytic vesicle, pore complex, recycling endosome |
| 111133628 | proton channel OtopLc-like | NA | monoatomic cation transport, cGMP biosynthetic process, Golgi to endosome transport | NA |
| 111134691 | NAD(+) hydrolase SARM1-like | proton channel activity, channel activity | signal transduction, receptor guanylyl cyclase signaling pathway, G protein-coupled receptor signaling pathway | membrane, dendrite |
| 111135424 | type-1 angiotensin II receptor-associated protein-like | NA | protein phosphorylation, proteolysis | NA |
| 111136759 | DELTA-actitoxin-Afr1c-like | signaling adaptor activity | negative regulation of MyD88-independent toll-like receptor signaling pathway, intracellular signal transduction, angiotensin-activated signaling pathway, defense response to bacterium | NA |
| 111125809 | tektin-like protein 1 | NA | NA | NA |
| 111125808 | uncharacterized LOC111125808 | NA | NA | NA |

**Supplementary Table 2.** Descriptive names and GO terms for genes adjacent to SNPs associated with survival in GCF_053477285.1. The column “gene name” corresponds to the “description” data in the GFF annotation file provided with the RefSeq release.

| Component | PBLUP | GBLUP |
| --- | --- | --- |
| Direct genetic effect | .0311 | .0213 |
| Common environment effect | .00583 | .00763 |
| Residual | .165 | .171 |

**Supplementary Table 3.** Estimated variance components for the animal model with either the pedigree-based (PBLUP) or genomic (GBLUP) relationship matrix and all phenotypes (no masked samples).
